# Physico-Chemical and Antimicrobial Evaluation of Ozonated Olive Oil Produced with a Medical-Grade Generator for Veterinary Purposes

**DOI:** 10.3390/microorganisms13081932

**Published:** 2025-08-18

**Authors:** Călin Cosmin Repciuc, Giulia-Ana-Maria Vișan, Bernadette-Emoke Teleky, Adela Pintea, Cristiana Ștefania Novac, Nicușor Valentin Oros

**Affiliations:** 1 Department of Surgery Anesthesiology and Intensive Care, Faculty of Veterinary Medicine, University of Agricultural Sciences and Veterinary Medicine, 400372 Cluj-Napoca, Romania; giulia.visan@usamvcluj.ro; 2 Institute of Life Sciences, Faculty of Food Science and Technology, University of Agricultural Sciences and Veterinary Medicine, Calea Mănăștur 3-5, 400372 Cluj-Napoca, Romania; bernadette.teleky@usamvcluj.ro; 3 Department of Biochemistry, Faculty of Veterinary Medicine, University of Agricultural Sciences and Veterinary Medicine, 400372 Cluj-Napoca, Romania; apintea@usamvcluj.ro; 4 Department of Microbiology, Faculty of Veterinary Medicine, University of Agricultural Sciences and Veterinary Medicine, 400372 Cluj-Napoca, Romania; cristiana.novac@usamvcluj.ro; 5 Department of Internal Medicine, Faculty of Veterinary Medicine, University of Agricultural Sciences and Veterinary Medicine, 400372 Cluj-Napoca, Romania; nicusor-valentin.oros@usamvcluj.ro

**Keywords:** ozonated oil, peroxide, antibacterial, antifungal, wounds

## Abstract

The search for broad-spectrum antimicrobial products that do not generate resistance upon multiple applications has led to increased scientific and clinical interest in ozonated oils. The aim of this preliminary study was to evaluate the physico-chemical structure and antimicrobial properties of 1–12 h ozonated extra virgin olive oil produced in a veterinary clinic with a medical-grade generator. Prolonging the ozonation time causes a decrease in the iodine index, followed by significant increases in viscosity, acidity index, and peroxide values (*p* < 0.001). Other similar studies using industrial generators obtained satisfactory clinical results at peroxide values between 335 and 3590 mEq O_2_/Kg. Contrary to these established minimum thresholds, we found that ozonated olive oil with a peroxide index of 184 and 224 mEq O_2_/Kg exhibits fungicidal and bactericidal effects, demonstrating significant differences (*p* < 0.05) between tested and control samples for strains such as *Staphylococcus aureus*, *E. faecalis*, and *E. coli*. The 12 h ozonated oil showed itself to be efficient in the treatment of a 3-year-old cat presenting a chronic infected wound. The results encourage more detailed investigations of the antimicrobial effect of ozonated oils obtained with medical-grade generators and their evaluation on bacterial strains isolated from different individuals, followed by clinical evaluations and standardization.

## 1. Introduction

Local bacterial superinfections are among the most common complications in skin lesions with impaired healing. Because antibiotic resistance has become a major concern in recent years, research has been conducted into alternative treatment options, including ozone-based products [1,2,3,4,5,6,7,8,9,10,11,12,13,14,15,16,17,18,19,20,21]. Research has focused on finding products with antiseptic and antimicrobial potential, structurally different from classic antibiotics, effective against microorganisms, without the risk of generating resistance after multiple applications, and having minimal or no side effects. For these purposes, ozonated products prove themselves to be reliable agents [9,10,11,12,13,14,15,16,17,18,19,20,21].

Ozone is an allotropic form of oxygen, composed of three oxygen atoms and is a colorless gas that forms naturally in the atmosphere through the action of ultraviolet radiation on molecular oxygen [1].

There are several hypotheses regarding the bactericidal effect of ozone, but the most widely accepted theory seems to be that the main action occurs at the level of the cell wall, by affecting its integrity due to peroxidation reactions of phospholipids and lipoproteins [1]. Consecutively to these reactions, changes initially occur in the membrane structure that interfere with the exchange of metabolic products between the cell and the external environment, and in the final stage, the disintegration of the membrane and the release of cell components into the extracellular environment take place [1]. Rangel et al. (2022) reported that Gram-positive cocci, specifically *S. aureus* and *S. enterica*, exhibited greater resistance to gaseous ozone compared to the Gram-negative strains *E. coli*, *P. aeruginosa*, and *Acinetobacter baumannii*. Following exposure to 1.4 ppm of ozone for 10 h, reductions in bacterial growth were observed at 30% for *E. coli*, 25% for *P. aeruginosa*, and 15% for *A. baumannii*, while *S. aureus* and *S. enterica* showed no significant changes relative to controls. These contrasting outcomes underscore a gap in the current literature regarding the differential susceptibility of Gram-positive and Gram-negative bacteria to gaseous ozone disinfection, emphasizing the need for further research to clarify these dynamics [22].

Although gaseous ozone has recently attracted significant research interest, a closer examination reveals the absence of standardized methodologies specifically designed for evaluating its effectiveness as a surface disinfectant. This highlights the need to adapt existing testing standards to accommodate the unique properties of gaseous ozone [23]. As noted in the broader context of ozone’s antimicrobial properties, the use of ozone in veterinary medicine is similarly characterized by a lack of standardization and a predominantly empirical approach. This is underlined by the findings of Orlandin et al. (2021), who reported that approximately 20% of published studies in the field failed to provide essential data, highlighting inconsistencies in methodology and reporting within veterinary ozone research [24].

Ozonation of oils creates the possibility of producing formulations with ozone derivatives, which exhibit greater environmental stability. By improving storage capacity and reducing the toxicity that ozone has in gaseous form, a safer product that can also be used by owners is obtained, thus reducing the stress factors associated with bringing the patient to the clinic when possible [2]. Also, ozonated oils have antibacterial, anti-inflammatory, and antifungal properties like ozone used as such, but the mechanism of action is different, and additionally, oils offer the advantage of retaining these properties [3]. The oil’s properties of stabilizing ozone, in the form of ozonides, formed between the double bonds of polyunsaturated fatty acids (PUFAs), make the ozonation of oils a good option for preserving and delivering the ozone-beneficial effects to the skin level [3].

Ozonated oils exert their antimicrobial effects through multiple, well-documented mechanisms. Primarily, they induce oxidative damage by releasing ozonides and lipid peroxides that compromise microbial cell membranes, proteins, and nucleic acids, leading to cell death [11,25]. Additionally, these oils generate reactive oxygen species (ROS) such as singlet oxygen and peroxyl radicals, which penetrate microbial cells and cause oxidative stress, resulting in cytoplasmic leakage and membrane disintegration [6,11]. Their capacity to disrupt biofilms further enhances antimicrobial efficacy, as ozonated compounds degrade the extracellular polymeric substances, facilitating the inactivation of sessile bacterial populations [6,26]. Beyond direct antimicrobial action, ozonated oils also modulate host immunity by inducing the expression of antimicrobial peptides—such as S100A8 and S100A9—and stimulating phagocytic responses through lipid peroxidation products [11,26]. Together, these multifaceted actions make ozonated oils promising candidates for topical antimicrobial therapies, particularly in contexts involving resistant pathogens or chronic infections.

The objective of this study was to preliminarily develop and evaluate an autochthonous product based on ozonated olive oil, obtained with a medical-grade generator and its ozonation accessories available in the clinic hospital of the Veterinary Medicine faculty of Cluj-Napoca. An in vitro evaluation of the antibacterial effect of the ozonated olive oil was performed on different standardized bacterial and fungal cultures, as well as its physico-chemical analysis. Also, the comparison with the available similar studies and the evolution of a clinical case were among the objectives of the present study.

## 2. Materials and Methods

### 2.1. Ozonation Method of Olive Oil

Ozone was obtained using a medical generator (Medozon Compact; Herrmann Apparatebau GmbH, Elsenfeld, Germany) from medical oxygen. A quantity of 250 milliliters of extra virgin *Olea europaea* var Arbequina oil was introduced into a standardized glass fluid bubbler (Aquazon; Herrmann Apparatebau GmbH, Elsenfeld, Germany). The oil was bubbled at room temperature (22 °C) for 1, 3, 6, and 12 h at a constant ozone concentration of 80 μg/mL, this being the maximum concentration allowed by the generator and also agreed to be the highest usable for other ways of medical administrations. The oil temperature was not controlled during the ozonation process. The oxygen–ozone mixture flow rate was 4 L/min, this being the highest flow rate obtainable with the used generator. After the ozonated oil was obtained, it was kept at 4 °C until further physico-chemical and microbiological determinations were performed.

### 2.2. Physico-Chemical Analysis

The peroxide value, acidity, iodine, and viscosity were evaluated for the physico-chemical characterization of the products. All the chemicals and reagents were of analytical grade, purchased from Merck (Darmstadt, Germany) and Sigma-Aldrich (Steinheim, Germany). Ultrapure water was obtained using a Milli-Q water purification system.

#### 2.2.1. Peroxide Value

The official titrimetric AOCS method was used for the determination of peroxide value (AOCS, 1998) [27]. In an Erlenmeyer flask, 1 g of sample was weighed, 20 mL of a 2:1 mixture of glacial acetic acid (99%) and chloroform (99%) was added, followed by 1 mL of a saturated 99.5% potassium iodide (KI) solution. The sample is shaken until complete dissolution and then let stand in the dark for 3 min at room temperature. In parallel, a blank sample was prepared.

After this interval, 20 mL of distilled water was added, and titration with sodium thiosulfate 0.01 N was performed until a light-yellow color was obtained. At this point, 0.5 mL of 1% starch solution was added, which caused the color to change to dark blue. Afterward, the sample was titrated again until complete discoloration.

The peroxide value represents the amount of peroxide, measured in milliequivalents of active oxygen contained in 1000 g of sample (mEq O_2_/Kg), calculated using the following formula:

(1) $$\mathrm{Peroxide}\;\mathrm{Value}=\;\frac{(v1-v0)\times c\times 1000}{m}$$

where v1 represents the volume of sodium thiosulfate solution used for titration, v0 is the volume of sodium thiosulfate solution used for the blank sample, c is the concentration of the sodium thiosulfate solution, and m represents the amount of sample subjected to determination.

#### 2.2.2. Acid Value

Acid value was determined in accordance with AOCS Official Method Ca 6b-53. In an Erlenmeyer flask, 1 g of sample was introduced along with 20 mL of a 1:2 mixture of ethanol (96%) and diethyl ether (99.97%). Then, 2–3 drops of phenolphthalein were added. With continuous stirring, they were titrated with 0.1 N potassium hydroxide (KOH) (95%) until the equivalence point (a pink coloration that persists for 1 min).

The acidity value represents the amount of potassium hydroxide needed to neutralize the free fatty acids contained in one gram of sample (mg KOH/g), calculated using the following formula:

Acid value = 56.1 × n/m,(2)

where n represents the volume of potassium hydroxide used for titration, and m represents the amount of sample subjected to determination.

#### 2.2.3. Iodine Value

Iodine value was determined according to the AOCS: Cd 1-25, Cd 1b-87, and Cd 1d-92 method. An Erlenmeyer flask, perfectly clean and dry, was weighed. The sample to be analyzed was added, and the flask was reweighed. The difference between the two measurements represented the sample mass, with its quantity being inversely proportional to the expected iodine value (0.1–0.8 g), and 15 mL of chloroform was added. The blank sample consisted only of 15 mL of chloroform.

Then, in both samples, 15 mL of Hanus solution (iodine concentration of 2.1–2.5%) was added to both the sample and the control. The flasks were stoppered, shaken, and left in the dark for 15 min.

At the end of this interval, 10 mL of KI was added to each, shaken vigorously, and titrated with a sodium thiosulfate solution. Toward the end of the titration, when the solution’s color lightened to yellow, 1 mL of starch was added, and titration continued until the complete disappearance of the dark brown color of the complex formed between iodine and starch

The iodine value represents the amount of iodine that reacts with the double bonds present in 100 g (g/100 g) of sample, calculated using the following formula:

Iodine Value = (n1 − n2) × 12.69/m,

where n1 is the amount of sodium thiosulfate solution used for the blank sample, n2 is the volume of sodium thiosulfate solution used for the sample subjected to determination, and m represents the amount of sample subjected to determination.

Each measurement was performed in triplicate, and the mean values **±** Standard Deviation were presented.

#### 2.2.4. Viscosity Determination

The rheological analysis of the olive oil, both before and after treatment, was conducted using an Anton Paar MCR 72 rheometer (Anton Paar, Graz, Austria). This rheometer was equipped with a Peltier plate–plate system (P-PTD 200/Air, Anton Paar, Graz, Austria) featuring temperature control and a smooth parallel plate geometry (PP-50-67300, Anton Paar, Graz, Austria) with a 50 mm diameter [6]. Viscosity curves were measured at various shear rates ranging from 5 to 300 s^−1^, employing logarithmic stepwise increments. Approximately 3 mL of samples were directly applied to the lower plate and tested at two temperatures (22 and 35 °C). The gap between the plates was set at 1 mm, and any excess sample was removed before each measurement. Each measurement was performed in triplicate, and the mean values are presented.

#### 2.2.5. Antimicrobial Activity

Four samples of ozonated extra virgin olive oil, ozonated for different periods of time (1, 3, 6, and 12 h, respectively), and a control sample of non-ozonated oil were tested. Reference strains of fungi used for evaluation included *Candida albicans* DMSZ 1386; Gram-positive bacteria *Staphylococcus aureus* ATCC 6538P and *Enterococcus faecalis* ATCC 29212; and Gram-negative bacteria *Escherichia coli* ATCC 13076, *Pseudomonas aeruginosa* ATCC 27853, and *Klebsiella pneumoniae* NCTC 13438. Bacteria were cultured on Nutrient Agar (NA) medium, and Sabouraud Dextrose (SAB) medium (Merck, Darmstadt, Germany) with added chloramphenicol was used for *Candida albicans*. All strains used were provided by the Microbiology Department of the Faculty of Veterinary Medicine within USAMV Cluj-Napoca.

#### 2.2.6. Diffusimetric Method Protocol

For this study, a modified EUCAST protocol [28] was used as follows: in sterile Petri dishes, 20 mL of Mueller Hinton agar (Merck, Darmstadt, Germany) was poured, which was subsequently allowed to solidify and dry at 37 °C for 30 min. Cultures of the reference strains, incubated overnight, were diluted in sterile physiological saline (0.9% NaCl, Sigma Aldrich, Darmstadt, Germany) to an optical density of 0.5 on the McFarland scale, a value established with an optical densitometer (Biosan, Riga, Latvia). The strains were inoculated on the surface of culture media using sterile cotton swabs and the three-section technique.

Due to the diffusion difficulties of the oil in agar, a Tween 80 solution was used to facilitate the emulsification of the oil and its diffusion on the surface of the culture medium. The concentrations of Tween 80 used were 0.5 and 2.5%, and its dilution was made in dimethyl sulfoxide solution [29]. Oils emulsified with Tween 80 at a concentration of 2.5% showed themselves to be more effective in the case of some bacterial strains like *Staphylococcus aureus*, increasing the diffusion capacity, as also suggested by Hood et al. in 2003 [29].

Also, for these reasons, two methods were used to carry out the microbial activity testing: the well diffusion test and the filter paper disc diffusion test, as proposed by Hood et al., 2003 [29]. A total of 10 µL of oil was applied to the filter papers, and 60 µL was used for the wells. The negative control was represented by the Tween 80 solution, and for the positive control, an antibiotic micro tablet with good effects on the targeted bacteria was chosen: G+ Amoxicillin (AX 10 µg), G- Gentamicin (GEN 30 µg), fungi Ketoconazole (10 µg). The commercial antibiotic and antifungal disks were purchased from Liofilchem, Teramo, Italy. All cultures were incubated at 37 °C for 24 h, and subsequently, the diameters of the inhibition zones were measured using a digital caliper. All determinations were performed in triplicate.

#### 2.2.7. Minimum Inhibitory Concentration (MIC)

The results obtained from the diffusion test showed that the sample ozonated for 12 h had the highest antibacterial capacity, which is why it was decided to test the minimum inhibitory concentration only for this sample in order to observe the lowest concentration of oil that can inhibit the growth of the tested bacteria and yeasts.

On the day before the determination, the cultures were subcultured on NA in the case of bacteria and on SAB medium for the *Candida albicans* strain. After their incubation at 37 °C for 12 h, a dilution in broth was made to a density of 0.5 on the McFarland scale for bacteria and 1 for fungi. Incubation was carried out in a shaking incubator (Orbital Shaker Incubator, Biosan, Riga, Latvia), under the same conditions used for cultivation.

After this interval, the Stock solution was prepared in sterile 2 mL Eppendorf tubes by emulsifying the oil with a 10% Tween 80 solution, the ratio between the two being 1/10. Subsequently, serial dilutions were made starting from equal amounts (500 µL) of the Stock solution and sterile nutrient broth (1/2) up to a ratio of 1/8 between the two, and 500 µL of bacterial culture was added to each tube. Four negative control tubes were also prepared, containing, in turn, Stock solution, oil and 10% Tween 80 diluted in broth, and broth alone. The positive control was represented by the bacterial and yeast cultures in the nutrient broth. Incubation was carried out in a shaking incubator at 37 °C for 24 h, and subsequently, 500 µL of each suspension was inoculated into Petri dishes with nutrient agar, using a flamed and flame-molded glass spreader. The plates were left to incubate overnight at 37 °C, and the colonies were counted the following morning.

In cases where the colonies could not be counted at the obtained product dilutions (1/4, 1/8, 1/16), successive 10-fold dilutions were made for the 1/4 dilution, followed by incubation and reading after 24 h.

#### 2.2.8. Clinical Case

**Figure 1:**
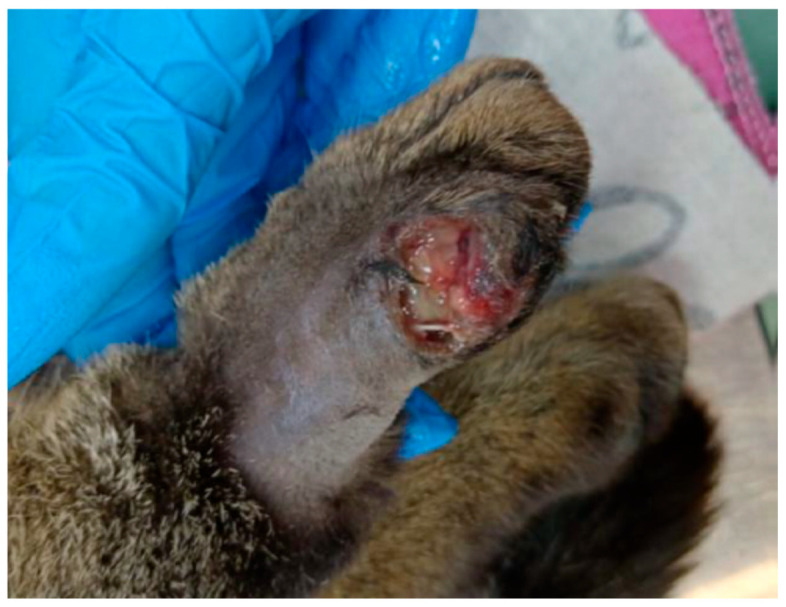
Wound of the patient showing purulent secretions, atonic borders with portions of necrotic tissue, and poor overall vascularization.

A 3-year-old male feline patient was presented to the Veterinary Emergency Hospital of the Faculty of Veterinary Medicine at the University of Agricultural Sciences and Veterinary Medicine in Cluj-Napoca with a non-healing, superinfected wound located on the lateral side of the right hind limb’s fourth digit (Figure 1).

Before initiating wound cleaning, samples were collected for microbiological examination and antibiotic susceptibility testing. The results revealed the presence of *Staphylococcus* spp. and *Pasteurella* spp. strains, which were found to be sensitive to most of the antibiotics tested (Amoxicillin, Ceftiofur, Trimethoprim/STX, Doxycycline, Gentamicin, Cefotaxime), showing resistance only to Clindamycin.

With the owner’s consent, treatment was initiated using topical applications of 12 h ozonated oil, without the administration of antibiotics. The care protocol included cleansing the wound, rinsing it with saline solution, drying with sterile gauze, applying the ozonated oil, and protecting the area with a bandage consisting of non-adherent dressings, a thin layer of orthopedic cotton, and VetWrap. The ozonated oil was applied daily until complete healing was achieved.

#### 2.2.9. Statistical Analysis

All statistical analyses were conducted using Python v3.11 software with the SciPy v1.11.4, pandas v2.1.4, and numpy v1.26.0 libraries. For the microbiological determinations, paired and unpaired Student’s *t*-tests were performed using the ttest_rel and ttest_ind functions from the scipy.stats module to compare inhibition zone diameters between ozonated oil treatments and their corresponding control. The Kruskal–Wallis test was applied using the kruskal function in small sample sets. Linear regression analyses were conducted with the linregress function to evaluate intra-group variability across sample triplicate values. All data preprocessing and structuring were performed using the pandas data analysis library.

For the physico-chemical determination values, one-way ANOVA was performed using the ols and anova_lm functions from the statsmodels library to evaluate the impact of ozonation time on each parameter. Where applicable, post hoc Tukey’s HSD tests were performed for multiple comparisons.

Correlation analysis was conducted to evaluate the relationship between the physico-chemical properties of 6 and 12 h ozonated oils and their antifungal activity against *Candida albicans*. Inhibition zone diameters obtained using the filter paper diffusion method and the 2 different Tween 80 concentrations were compared to peroxide value, acidity, iodine index, and viscosity at 22 °C and 35 °C. Pearson correlation coefficients were also calculated.

## 3. Results

### 3.1. Physico-Chemical Analysis of Ozonated Oil

By dynamically tracking the results, it can be observed that the peroxide value and acidity increased while iodine values decreased proportionally with ozonization time (Table 1).

The linear regression analysis performed to evaluate the relationship between ozonation time and the physico-chemical properties of the oils demonstrated a strong, statistically significant positive correlation between ozonation time and peroxide value (*p* = 0.011) and acidity (*p* = 0.0012). These parameters increased consistently with longer exposure durations, indicating progressive oxidation and structural changes in the oil. Conversely, the iodine value showed a significant negative correlation with ozonation time (*p* = 0.0033), reflecting a decrease in unsaturation levels. Overall, these findings confirm a clear time-dependent effect of ozone exposure on the chemical composition of the oil samples.

Ozonation of oils leads to an increase in their viscosity and the loss of color as the ozonation time increases. The 12 h ozonated oil was the most viscous, showing a solid consistency at 5 °C and a 1640.5 mPas difference between the 22 °C and 35 °C tested samples, as shown in Table 1.

**Figure 2:**
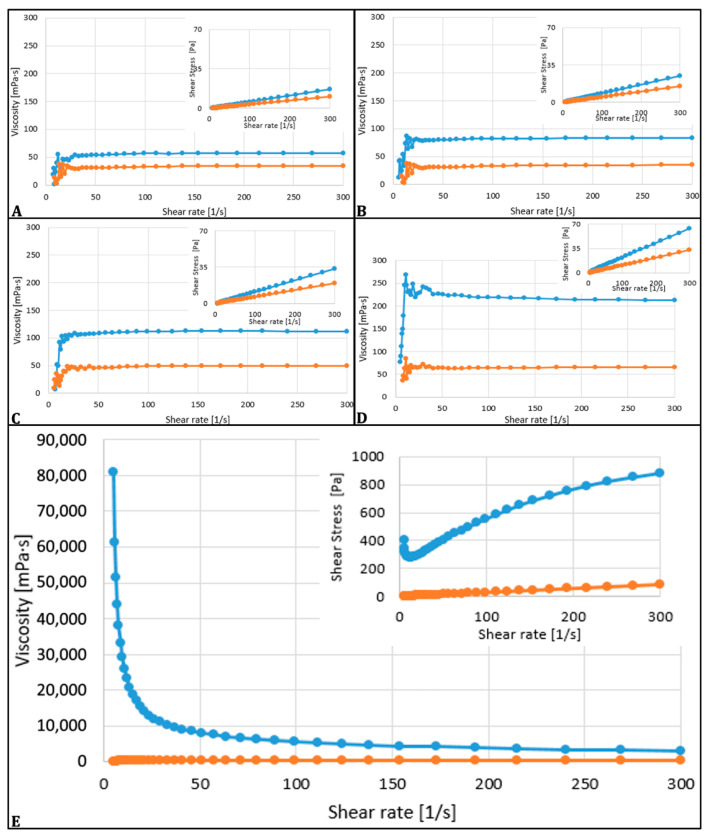
The rheological behavior expressed by viscosity curves of olive oil samples measured at 22 °C (blue) and 35 °C (orange) across a range of shear rates (5–300 s^−1^). (**A**). Unozonated olive oil (UN); (**B**). Olive oil ozonated for 1 h (UO1); (**C**). Ozonated for 3 h (UO3); (**D**). Ozonated for 6 h (UO6); (**E**). Ozonated for 12 h (UO12). *X*-axis: Shear rate (s^−1^); *Y*-axis: Viscosity (mPas). Each curve represents the mean viscosity from three replicate measurements at each temperature.

The rheological characteristics of ozonated olive oils were assessed across shear rates ranging from 5 to 300 s^−1^, illustrating the relationship between viscosity and shear rate, and can be depicted in Figure 2.

The unozonated olive oil and ozonated for 1–6 h presented a Newtonian behavior, while the olive oils treated with ozone for 12 h across the investigated range of shear rates exhibited pseudo-plastic (shear-thinning) behavior. As depicted in the figures, at both temperatures, the viscosity gradually declined with increasing shear rate, characteristic of biopolymer solutions.

The shift from Newtonian to pseudo-plastic (shear-thinning) behavior in the oil sample after 12 h of ozonation indicates a significant alteration in its chemical structure.

### 3.2. Antimicrobial Activity of Ozonated Oil

#### 3.2.1. Diffusimetric Method

The method of applying the oil to the agar influenced the dispersion of the oil in the agar and, consequently, the diameter of the inhibitory zone. As can be seen in Table 2, for Gram-negative bacteria, only the oil ozonated for 12 h from the wells produced an inhibitory effect. For the *Candida albicans* strain, the same oil sample applied in wells exceeded the inhibition zone of the ketoconazole micro tablet, and the one applied on filter paper had results close to it. The mean diameter of the inhibition zones produced by the ozonated oil was 20.35 mm, slightly higher than the 19.55 mm observed for the antifungal control. However, this difference of 0.8 mm was not statistically significant (*p* = 0.6694). These findings suggest that while ozonated oil demonstrates inhibitory activity against *Candida albicans*, it has similar efficacy to the conventional antifungal treatment under the tested conditions.

The Gram-positive bacteria tested samples applied in wells showed an inhibitory effect, and the read values were directly proportional to the ozonation time of the sample.

The analysis of antimicrobial inhibition zones revealed that in most cases, conventional antibiotics produced significantly larger inhibition zones compared with the ozonated samples. Paired *t*-test demonstrated significant differences between ozonated oils and control samples for strains such as Staphylococcus aureus, *E. faecalis*, and *E. coli* (*p* < 0.05). Kruskal–Wallis tests supported these results, confirming the statistical relevance of the findings. For the Gram-negative strains, UO12 effects were lower than the antibiotic inhibition zones, confirming significantly lower efficacy (*p* < 0.001). Candida albicans treated with UO12 had inhibition zones that did not significantly differ from those of the antimycotic product tested, showing that 12 h ozonated oil has potential antifungal utility (*p* > 0.05). Linear regression in all replicates showed strong internal consistency.

The correlation analysis performed showed that higher peroxide, acidity, and viscosity were associated positively with greater diameter inhibition zones for Candida albicans (r = +0.679); meanwhile, the iodine index showed a moderate inverse relationship with the antifungal positive effect, suggesting a good antifungal effect and a satisfactory saturation of the oil samples.

Summarizing the microbiological results, we can assess the strain-specific and condition-dependent effects of the tested oil samples with evident antifungal potential and moderate gram-positive antibacterial activity.

#### 3.2.2. Minimum Inhibitory Concentration

By diluting the ozonated oil sample in sterile nutrient broth, satisfactory results were obtained regarding the minimum oil concentration required to inhibit the growth of the tested yeasts and some bacterial strains. The best inhibitory effect of the tested oil was demonstrated on the *Candida albicans* strain, where the sample diluted to a concentration of 1.25% had a 100% inhibitory effect. The same effect was obtained for the *S. aureus* and *K. pneumoniae* strains when the oil was diluted to 2.5%. No inhibitory effects were obtained upon dilution of the oil sample for the *E. faecalis*, *E. coli*, and *P. aeruginosa* strains. The next step was to perform successive dilutions for both the 1/4 concentration tube and the positive controls of each strain. Colony counting was impossible, concluding the necessity of using an undiluted sample to obtain a positive inhibitory effect. All results obtained in this stage of antimicrobial activity testing are detailed in Table 3.

**Figure 3:**
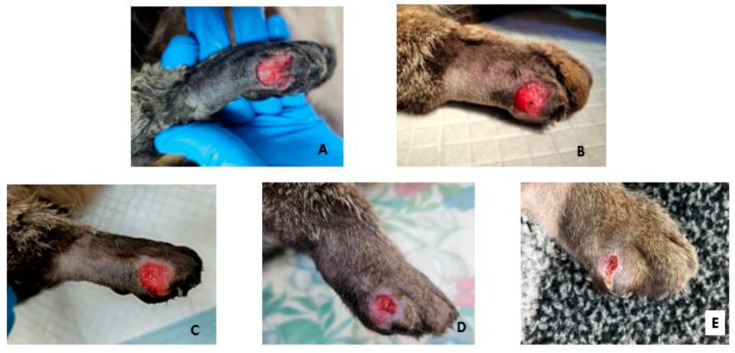
Wound healing appearance: (**A**)—Day 2, (**B**)—Day 5, (**C**)—Day 8, (**D**)—Day 16, (**E**)—Day 19.

The patient showed a favorable progression, with healing occurring rapidly and comfortably. At the first bandage change, natural debridement was observed, along with the presence of early granulation tissue (Figure 3A). At the next evaluation on day 5, granulation tissue covered the entire wound surface (Figure 3B), and by day 8, the epithelialization process had begun (Figure 3C). Wound contraction reached 50% of the initial surface area by day 16 (Figure 3D). Epithelialization was 95% complete by day 19 (Figure 3E).

## 4. Discussions

In veterinary medical practice, the method of ozone administration is chosen based on the type and location of the treated condition. Because each method has specific effects and benefits, combining them can create a synergistic action that enhances more beneficial effects for the patient. This allows us to create a personalized protocol that can increase therapeutic efficacy.

Ozonated oils have been widely used topically for their potent antibacterial properties in the treatment of various conditions. It has been hypothesized that their effectiveness in wound healing is primarily due to their strong antimicrobial activity, complemented by their ability to stimulate the production of growth factors, activate local antioxidant mechanisms, and promote tissue regeneration [9]. The ozonolysis of fatty acids after the reaction of oils with ozone takes place at the level of the double bonds of unsaturated fatty acids, producing firstly unstable ozonides, which further decompose to form aldehydes and highly reactive Criegee intermediates (zwitterion). In anhydrous environment, carbonyl compounds will combine to generate cyclic trioxolane [30]. Finally, a wide range of products is obtained, such as hydroperoxides, ozonides, aldehydes, peroxides (including di- and polyperoxides), which are considered to be responsible for the antibacterial and fungicidal activity of ozonated oils [3,31]. Ozonization of vegetable oils is accompanied by an increase in the PV with the ozonation time, and a reduction in iodine index was obtained in our study as well. Moreover, the addition of water significantly increases the formation of peroxides, thus the increase of PV [14,32]. Our results indicate that during the process, through the synthesis of ozonides, the double bonds in the oil’s structure react with ozone molecules, generating reactive oxygen species. This increases the peroxide value and the solution’s acidity [5]. Thus, we can conclude that the number of double bonds in the oil’s structure, and consequently the quantity of 1,2,4-trioxolane generated, influences the amount of peroxide formed during the process. In practice, this determination is used to assess the stability of the ozonated oil obtained and as a storage control method [6]. Furthermore, the quantity of peroxide produced can be considered an indicator of the sample’s antibacterial capacity [7]. In our study, PV of ozonized samples was determined according to the standardized iodometric assay. Although this is still the most commonly used method, recent studies emphasized the need for optimization. Among the parameters that could be optimized, one can mention the temperature, the reaction time, the potassium iodide, or the amount of sample. Using an optimized method, Jacinto et al. (2024) obtained higher PV values and better discrimination between different ozonized oils [33].

The peroxide value (PV) is a common method to determine the primary oxidation products of lipids, mainly hydroperoxides. Lipid hydroperoxides further decompose to form secondary oxidation products, including aldehydes, ketones, epoxides, alcohols, organic acids, cyclic peroxides, and polymeric products [34,35]. Several methods have been developed for the determination of PV of vegetable oils, including ozonated oils used for medical applications. The most common method to determine PV is the iodometric determination of peroxides, by titration, colorimetric, or electrometric methods [32,34]. Volumetric measurement of PV is based on the ability of hydroperoxides to oxidize the iodide ion of potassium iodide to iodine, which is further titrated with a standardized solution of sodium thiosulfate, using starch as an endpoint indicator [35,36]. The volumetric method is an empirical one, and the results might be affected by interferences with oxygen, reaction time, light exposure, or temperature [33,34]. A limitation of this method is that only hydroperoxides that liberate iodine in acid solution can be determined, while cyclic peroxides, polymeric peroxides and other non-iodine-liberating products formed during lipid oxidation cannot be measured or will be underestimated [33,37]. Other, more sensitive spectrophotometric (Ferric Thiocyanate Method) or spectroscopic methods, like Mid and Near Infrared Spectroscopy (MIR and NIR), have also been used to evaluate the PV of crude or ozonated oils [32,38].

The ozonation of oils influences their physico-chemical and rheological properties, especially if an extended exposure time is used. The most relevant of all was the increase in viscosity with prolonged ozonation. The phenomena are attributed to the formation of polymeric peroxides and other high molecular weight oxidative compounds. These elements are known as being responsible for the thickening process of oil matrix and its flowability reduction at lower temperatures, as reported by Uysal in 2014 [8]. In our study, the 12 h ozone-exposed oil had the highest viscosity, showing solid consistency at 5 °C refrigeration time and a significant difference of 1640.5 mPas between the two measured temperatures (22 °C and 35 °C), showing high statistical relevance (*p* < 0.001). The oils commonly available in the market exhibit characteristics of ideal Newtonian fluids, showing a rapid decrease in viscosity as temperature rises. The preset shear rate is determined by dividing the flow velocity of the sample by the shear gap diameter, which, in our case, is 1 mm. It can be noted that the viscosity is influenced by temperature, indicating that for each sample analyzed, viscosity significantly decreased as temperature rose [39]. The presented findings are similar, with the ones in another report explaining the temperature–viscosity relationship in oils, viscosity decreasing with the increase of temperature, phenomenon that happens due to intermolecular interactions breakdown [39]. Nevertheless, the rheological behavior of the analyzed samples was evaluated across a shear rate range of 5 to 300 s^−1^. Newtonian characteristics were displayed by the unozonized sample and the 1, 3, and 6 h ozonated samples. The 12 h sample manifested a distinct pseudo-plastic behavior. The pseudo-plastic behavior results in reduced shear viscosity at higher shear rates due to the alignment of large molecular chains, leading to lower resistance. This kind of behavior is indicative of biopolymeric fluids, where the share rate increases and viscosity decreases due to the alignment of long molecular chains, reducing the flow resistance [40]. The pseudo-plastic nature of the 12 h ozonized oil became more pronounced at both tested temperatures, suggesting the formation of high molecular weight compounds, such as ozonides, 1,2,4-trioxolanes and polymeric peroxides, which create a robust internal network in the oil [41]. This network hinders flow at low shear rates but allows easier flow at high shear rates, characteristic of pseudo-plastic fluids. This change highlights the substantial impact of oxidative conditions on the oil’s microstructure [41].

It is also important to understand how ozone directly reacts with the body’s fluids. Ozone generates multiple effects on cells, this being the starting point for achieving the desired therapeutic effect. The gaseous structure of ozone allows it to dissolve completely and easily in plasma and in the water of extracellular fluids or in the hydrolic film covering the mucous membranes. Through the action of secondary messengers, the body’s antioxidant systems are activated [42]. This is possible due to the moderate oxidative stress that induces the activation of nuclear transcription factors, such as nuclear factor erythroid 2-related factor 2, hypoxia-inducible factor 1-alpha, nuclear factor of activated T-cells, and activator protein-1 [42,43]. Due to its high oxidation potential, ozone has a rapid action on microorganisms, producing a high index of lethality against them. This effect occurs in two stages that take place simultaneously. The first involves the direct reactions of molecular ozone, and the second is centered on the destructive effect of the free radicals formed. The inactivation of bacteria by the action of ozone is a complex process, as ozone reacts with multiple cellular constituents, including proteins, unsaturated lipids, respiratory enzymes in cell membranes, peptidoglycan in the cell wall, enzymes, and nucleic acids in the cytoplasm [44].

Contrary to its interaction with the body fluids, Sadowska et al. in 2008 mentioned that the reaction of ozone with vegetable oils occurs almost exclusively with carbon–carbon double bonds present in unsaturated fatty chains, and some of the main reaction compounds are represented by ozonides [45]. De Almeida Kogawa et al., in 2015, also described that ozonation of vegetable oils leads to the generation of stable compounds like ozonides, which profoundly influence the physicochemical properties of the oil, including viscosity and thermal behavior [46]. A fundamental structural transformation in the oil sample matrix is highlighted by the transition from Newtonian to non-Newtonian flow induced by oxidative stress. This process is of significant interest in medical applications where product formulation and delivery are influenced by rheological properties. The ability of the ozonation process to influence the viscosity suggests a potential tailor-based system for antimicrobial treatments of topical use or other formulations with slow-release properties where shear responsiveness and viscosity control are of great interest [47,48].

Puxeddu et al., in 2024, evaluated two commercial products based on ozonated oil, obtained similar results regarding the antibacterial and antifungal effect of olive oil, with the mention that the respective product had a peroxide index value of 3110 mEq O_2_/Kg [10]. Other studies that evaluated the antibacterial/antifungal activity of ozonated oil-based products (Table 4) show variable results, most of them with a more pronounced effect at high peroxide index values (greater than 500 mEq O_2_/Kg). Skalska et al. suggested that the antibacterial activity is given by the amount of peroxide produced in the ozonation process [7]. For this reason, a directly proportional relationship is considered to exist between the peroxide index value and the biocidal capacity of the ozonated oil. The specialized literature generally recommends the use of an oil with higher peroxide index values when an antibacterial effect is desired. The use of ozonated oils with high peroxide index values may raise some questions regarding toxic effects, but studies have shown that ozone exhibits good cellular biocompatibility on human gingival fibroblasts [11]. All the above values are being compared for a better visualization in Table 4.

In our study, the physico-chemical data demonstrate that extended ozonation time has a significant influence. Peroxide and acidity values showed strong linear increases with time exposure, while the iodine index decreased sharply. The trends suggest a progressive oxidative enrichment of the oil samples, likely explainable by the formation of ozonides and peroxides. The high Pearson correlation between the mentioned parameters (r > 0.94) further suggests a coordinated chemical shift during ozonation. These transformations might explain the enhanced antimicrobial activity observed for the 12 h ozonized oil samples tested.

The ozonated oil demonstrated enhanced antifungal efficacy compared to gaseous ozone against dermatophytes from the genus *Microsporum—M. gypseum*, *M. canis*—by significantly reducing spore production, increasing cell membrane permeability, and inhibiting key hydrolytic enzymes involved in fungal virulence [26]. Also, a recent study demonstrates that ozonated extra-virgin olive oil (EOO) exhibits strong antifungal effects against *Candida albicans* by reducing cell viability, damaging cell walls, inducing oxidative stress and autophagy, and inhibiting hyphal and biofilm formation, while maintaining biocompatibility; similar effects were also observed against *Candida glabrata* [12].

From a bactericidal point of view, ozonated oil has a remarkable action against strains of *Streptococci*, *Enterococci*, *Staphylococci*, *Escherichia*, *Pseudomonas*, and especially against *Mycobacterium* spp. [13,14,15,16,17,18,19,20].

Regarding the diffusion method, Hood et al., in 2003, considered disc diffusion to be one of the most commonly used for evaluating the antibacterial activity of oils. Their study demonstrated a reduction in inhibition zones with increasing concentration of solubilizing agent, with the maximum value being 5% and the best results obtained by incorporating a 2.5% Tween 80 solution [29]. For these reasons, in the present study, we chose to use concentrations of 0.5 and 2.5%. No notable differences were observed between the samples when reading the inhibition zones.

In the study published by Moureu et al. in 2015, oil ozonization was performed both in the presence and absence of water, using an industrial-grade generator. Peroxide values varied significantly, being 397 mEq O_2_/Kg for the water-free oil sample and between 560 and 2680 mEq O_2_/Kg for the oil sample with water. The antimicrobial effect was observed only in samples ozonized in the presence of water. In the present study, the antimicrobial effect was evident from peroxide values of 118 mEq O_2_/Kg and increased proportionally with ozonation time. Comparing the two studies, we can observe that although the presence of water led to higher peroxide values and facilitated antimicrobial activity, this effect was also achieved in the current study without the use of water and at much lower peroxide values, obtainable through ozonation with a medical-grade generator [14].

Our results show that ozonated oil is less effective against Gram-negative bacteria, such as *Pseudomonas aeruginosa* and *Escherichia coli.* A possible hypothesis that could explain the resistance of *Ps. aeruginosa* could be the fact that this bacterium produces essential antioxidant enzymes (catalase, superoxide dismutase, peroxidases) to neutralize reactive oxygen species (ROS), thereby protecting itself from oxidative agents. *Pseudomonas aeruginosa* has two forms of superoxide dismutase (Fe-SOD and Mn-SOD) and three catalases (katA, katB, and katC), whose expression is regulated in response to stress. Moreover, biofilm formation increases tolerance to ROS [49].

For example, Gabriele Slavinskiene et al. did not experience a significant response difference to oils regarding bacterial strains, either gram-positive or negative. We cannot compare their results with ours because the paper does not mention the ozone concentration used for the 4 h ozonation time, nor does it mention the volume per minute [50].

Regarding *E. coli*, it is known that this bacterium expresses two cytoplasmic enzymes (Fe-SOD and Mn-SOD) and one periplasmic form (Cu/Zn-SOD). These enzymes neutralize the superoxide radical (O_2_^−^), keeping it at sub-nanomolar levels and thereby preventing damage to proteins and DNA. Enzymes such as Ahp (alkyl hydroperoxidase), catalase G (KatG), and catalase E (KatE) convert hydrogen peroxide (H_2_O_2_) into water. Ahp is active at moderate H_2_O_2_ concentrations, while the catalases take over under conditions of high oxidative stress [51]. These mechanisms provide *E. coli* with effective protection against reactive oxygen species, such as the ozonides found in ozonated oil. As a result, even if the oil generates ROS, the bacteria can rapidly neutralize these oxidative agents, thereby eliminating the antibacterial effect. Overall, the Gram-negative cell wall, with its outer membrane and lipopolysaccharides (LPSs), reduces permeability and provides natural protection against both hydrophobic and hydrophilic molecules, including oxidative compounds. In comparison, Gram-positive bacteria such as *Staphylococcus aureus* are more sensitive to ozonated oils, as the absence of an external membrane exposes them directly to oxidants. Other studies have also demonstrated that ozonated oils are more effective against Gram-positives, with weaker effects on Gram-negative microorganisms [52].

The antimycotic efficacy of the oil samples against *Candida albicans* appeared to be strongly influenced by their oxidative profile. The positive correlation between the inhibition zones and peroxide/viscozity suggests that formation of ozonides and peroxides during a period of time greater than 6 h contributes directly to antimicrobial efficacy. The inverse correlation with the iodine determination values is consistent with the decrease of unsaturated fatty acids, which are transformed during ozonation. Also, the superior efficacy observed for the 12 h ozonated sample against *Candida albicans* and some of the selected bacterial strains, supported by the clinical result presented, suggests that it could be integrated as a complementary topical antimicrobial agent in veterinary practice. Its activity, combined with a low risk for antimicrobial resistance development or chemical residue accumulation, makes UO12 an attractive option in the treatment of infected wounds in animals.

Phenolic compounds in olive oil, such as oleuropein, hydroxytyrosol, tyrosol, luteolin, apigenin, coumaric acid, and caffeic acid, possess significant antimicrobial properties against bacteria, including Escherichia. coli, Salmonella typhimurium, and Staphylococcus aureus, and fungi like Candida species [53,54,55]. These phenolics contribute not only to the antimicrobial effect but also to the antioxidant capacity and to the regenerative properties of olive oil [55]. Also, microbial inhibition in oils appears to depend on exposure duration. Ciafardini et al. (2013) reported that *Candida parapsilosis* can persist in extra virgin olive oil for up to 18 months, whereas Zullo et al. (2018) observed complete elimination of *Escherichia coli* within 15 days in olive oil containing a high level of 372 mg caffeic acid equivalents per kg [56,57]. These findings suggest that antimicrobial efficacy is shaped by the interaction between phenolic compounds levels, microbial susceptibility and environmental conditions. The absence of inhibition in our unozonized oil likely reflects insufficient phenolic compounds content and/or exposure time. In this context, it might be plausible that the antimicrobial activity observed in ozonated olive oil could be influenced by its phenolic content. Future investigations are warranted to elucidate the role of phenolic compounds in modulating the antimicrobial efficacy of ozonated olive oil and to better understand synergistic or additive effects.

Regarding its clinical efficacy, ozonated oil has been successfully reported to treat common eye disorders in horses and dogs [58,59,60]. Keratoconjunctivitis sica was also treated successfully with ozonated oil-based eye drops [61]. Dental issues have also been treated with ozonated oils. Abdelhamid, in 2012, used oleozon gel in the treatment of induced periodontal defects in mongrel dogs with very good effects [62].

Lenart-Boroń et al., in 2024, used an innovative formulation of ozonized olive oil in a hyaluronic acid matrix. The formula proved effective against 40.59% of the tested veterinary isolated bacteria, such as *Enterococcus* and *Acinetobacter* [63].

In an extensive study, 8 h ozonated olive oil obtained with an Ozonis steril 250 generator (250 mg of ozone per hour) was used for the treatment of mastitis in cattle. Promising results were observed against key mastitis-associated bacteria, suggesting its potential as an innovative approach to mastitis management [64]. Also, foot rot in sheep was successfully treated by using ozonated olive oil-derived ointments [65].

Aydin in 2022 used Face Ozonated olive oil (Face Ozon^®^, Ankara, Turkiye) exposed for 48 h to 1 g/h ozone. The study concluded that the use of ozone oil contributed to wound healing by a 50% quicker epithelialization [66].

Another study evaluating the effects of topical ozonated sunflower oil on second-intention healing of acute experimental skin wounds in red-eared sliders (*Trachemys scripta elegans*) showed that daily topical application over the course of one week improved the healing of acute, full-thickness skin wounds. The PV concentration of the oil was evaluated to 950 mEq O_2_/Kg [67]. Prządka, P. et al. in 2022, successfully treated a 3-year-old Labrador retriever with an extensive full-thickness skin laceration in the lumbar region by using gauzes soaked in commercial ozonated olive oil (Ozonella, Onkomed, Urzut, Poland), obtaining a complete epithelialization in about 35 days [68]. Another case report presenting a wound in a feline patient reported a complete epithelialization in 14 days of twice a day application of ozonated sunflower oil [69].

de Souza et al. showed an accelerated healing of the surgical wounds of 15 cats submitted to elective ovariohysterectomy [70]. Topical application of ozonated sunflower seed oil was highly effective in accelerating acute cutaneous wound repair, preventing hypergranulation tissue and infection in equine lower limb wounds in a study performed by Di Filippo et al. [71].

Gingival inflammation in cats was treated successfully with propolis extracted in ozone-enriched (5%) olive oil applied once a day for 14 d [72].

Ozonated oils seem to exert positive effects on other skin pathologies as well. A 650 mEq O_2_/Kg ozonated sunflower oil underscored complete inhibition of *Pythium insidiosum* isolated from dogs [73]. A dog with Pharmacodermia was successfully treated with the same product [74].

Commercial ozonated oils were cited to have also antiparasitic effects. Altinok Yipel et al. demonstrated that ozonated olive oil and *Origanum majorana* L. applied for 10 days of topical treatment achieved similar efficacy (>99%) as permethrin treatment (100%) against *Otodectes cynotis* (egg, juvenile, and adult forms) infestation in cats [75]. Also, Zamora Rodriguez et al. successfully treated giardiasis in dogs with 100% sunflower oil administered orally for 7 days, one week without treatment and another week of daily administration [76]. The same author managed to treat generalized demodicosis with ozonated oils, reporting that all the animals recovered the fur on more than 90% of the body surface and recovered from generalized demodicosis in 84 days of treatment, but the characteristics of the oil used are not presented [77]. Ozonated sunflower oil (Ozo3^®^) chemical compounds were efficient as anti-ticks (*Rhipicephalus linnaei*), altering the morpho-histology of the salivary glands and degenerating them early in rabbit models [78]. The same model was used by Vasquez Daud et al. to successfully show the efficacy of a commercial ozonated sunflower oil (Bioperoxoil^®^) against *Microsporum canis* [79].

In all the above-cited studies, commercially available products or industrially obtained ozonated oils were used. In our case, the 3-year-old patient was treated with the 12 h ozonated olive oil obtained with a medical grade generator, showing similar results at a lower peroxide value. The chronic, atonic, and infected wound of our patient healed completely in 19 days, the result being comparable to the one presented in a previously published case report [68].

## 5. Conclusions

The results obtained suggest that medical-grade ozone generators with a limited concentration output up to 80 μg/mL could be easily used in the production of ozonated olive oil with good in vitro effects.

The ozonated olive oil tested in this study had an antibacterial effect on standard bacterial cultures (*Staphylococcus aureus* ATCC 6538P, *Enterococcus faecalis* ATCC 29212, *Escherichia coli* ATCC 13076, *Pseudomonas aeruginosa* ATCC 27853, and *Klebsiella pneumoniae* NCTC 13438). The antibacterial effect was directly proportional to the peroxide value in the oil samples, which confirms that the peroxides and ozonides contained by ozonated oils are responsible for the antibacterial activity.

The antibacterial effect of the oil samples was observed starting from a low peroxide index value (119 mEq O_2_/Kg), with the best results obtained at a value of 224 mEq/1000 g, which corresponds to ozonating the oil for 12 h using the previously mentioned parameters. The antibacterial activity at this peroxide value was more pronounced on Gram-positive bacteria compared with Gram-negative bacteria.

The tested samples had a high antimycotic effect (*Candida albicans*), starting with a peroxide index value of 184 mEq O_2_/Kg. This result suggests that ozonated olive oils might be strong topic antimycotic products even at a lower peroxide index (184–224 mEq O_2_/Kg), in some cases, surpassing that of standard antimycotics. Also, our findings revealed a relevant relationship between the oxidative characteristics of ozonated oils and their antifungal activity, underlining the importance of ozonation time as one of the main factors in optimizing their antimicrobial efficacy.

The 12 h ozonated oil had favorable clinical results in the treatment of a chronic infected full-thickness digital wound in a 3-year-old male cat with a rapid 19-day healing process.

Considering the results of our study, we suggest that the integration of a medical-grade ozone generator made 12 h ozonated olive oil into the therapeutic management of chronically infected wounds in animals could be an effective and accessible treatment option for every veterinary clinic, making a good contribution to microbial resistance prevention.

Even if our results are encouraging, an extended study intending to standardize medical-grade generator oil ozonation protocols should also be conducted, comparing the properties of multiple vegetable oils, followed by clinical confirmation of the results, considering the different responses of each patient to standardized therapies would be of great interest.

## Figures and Tables

**Table 1:** Physico-chemical characterization of ozonized oil samples.

Sample	Peroxide Value (mEq O_2_/Kg)	Acidity Value (mg KOH/g)	Iodine Value (g/100 g)	Viscosity (mPas)	
UN	15.25 ± 0.02	0.53 ± 0.14	39.51 ± 0.89	22 °C	56.6
				35 °C	34.9
UO1	83.21 ± 0.06	2.55 ± 0.33	35.59 ± 1.1	22 °C	83.1
				35 °C	49.6
UO3	118.95 ± 0.10	2.90 ± 0.12	30.45 ± 0.7	22 °C	112.1
				35 °C	65.7
UO6	183.86 ± 0.20	4.54 ± 0.32	13.39 ± 0.3	22 °C	212.6
				35 °C	109
UO12	224.22 ± 0.22	8.31 ± 0.01	1.5 ± 0.01	22 °C	2937.3
				35 °C	296.8
	*p =* 0.011	*p =* 0.0012	*p =* 0.0033		-
					-

UN—non-ozonated oil, UO1—oil ozonated for 1 h, UO3—oil ozonated for 3 h, UO6—oil ozonated for 6 h, UO12—oil ozonated for 12 h, mEq O_2_—milliequivalent, KOH—potassium hydroxide, mPas—millipascal, g—gram.

**Table 2:** Antimicrobial activity—results of the diffusion method on different bacterial strains expressed as mean ± SD.

Microorganism		Tween 80 Concentration	The Diffusimetric Method	Inhibition Zone Diameter (mm)						
				Untreated Oil	Oil O_3_ 1 h	Oil O_3_ 3 h	Oil O_3_ 6 h	Oil O_3_ 12 h	Antibiotic/ Antimicotic	Negativ Sample
Gram-positive strains	*Staphylococcus aureus* ATCC 6538P	0.5%	Filter paper	0	0	0	6.5 ± 0.19	9.1 ± 0.27	13.3 ± 0.24	0
			well	0	0	4.1 ± 0.21	7.3 ± 0.32	10.4 ± 0.41	13.4 ± 0.12	0
		2.5%	Filter paper	0	0	0	0	10.9 ± 0.37	14 ± 0.32	0
			well	0	0	5.3 ± 0.13	7.9 ± 0.41	12.3 ± 0.52	13.9 ± 0.21	0
	*Enterococcus faecalis* ATCC 29212	0.5%	Filter paper	0	0	0	0	0	13.7 ± 0.12	0
			well	0	0	0	0	2.6 ± 0.11	13.2 ± 0.38	0
		2.5%	Filter paper	0	0	0	0	0	13.9 ± 0.26	0
			well	0	0	0	0	3.5 ± 0.17	13.5 ± 0.15	0
Gram-negative strains	*Escherichia coli* ATCC 13076	0.5%	Filter paper	0	0	0	0	0	19.7 ± 0.18	0
			well	0	0	0	0	7.5 ± 0.37	19.3 ± 0.23	0
		2.5%	Filter paper	0	0	0	0	0	20.7 ± 0.33	0
			well	0	0	0	0	9.5 ± 0.32	20.7 ± 0.13	0
	*Pseudomonas aeruginosa* ATCC 27853	0.5%	Filter paper	0	0	0	0	3.3 ± 0.14	25.2 ± 0.21	0
			well	0	0	0	0	0	24.9 ± 0.31	0
		2.5%	Filter paper	0	0	0	0	0	24.9 ± 0.17	0
			well	0	0	0	0	0	24.6 ± 0.14	0
	*Klebsiella pneumoniae* NCTC 13438	0.5%	Filter paper	0	0	0	0	0	16.7 ± 0.26	0
			well	0	0	0	0	7.9 ± 0.34	15.8 ± 0.32	0
		2.5%	Filter paper	0	0	0	0	0	16.7 ± 0.11	0
			well	0	0	2.8 ± 0.16	3.6 ± 0.14	8.6 ± 0.39	16.1 ± 0.16	0
Fungi	*Candida albicans* DMSZ 1386	0.5%	Filter paper	0	0	0	14.8 ± 0.48	16.6 ± 0.51	19 ± 0.32	0
			well	0	0	0	15.9 ± 0.36	23.3 ± 0.44	20.2 ± 0.19	0
		2.5%	Filter paper	0	0	0	0	17.6 ± 0.36	19.4 ± 0.24	0
			well	0	0	0	13.9 ± 0.13	23.9 ± 0.53	19.6 ± 0.36	0

**Table 3:** Antimicrobial activity—Minimal Inhibitory Concentration.

Micro-Organism		Dilution				
		1/4			1/8	1/16
		1/10	1/100	1/1000		
Gram-positive strains	*Staphylococcus aureus* ATCC 6538P	+	+	+	−	−
	*Enterococcus faecalis* ATCC 29212	−	−	−	−	−
Gram-negative strains	*Escherichia coli* ATCC 13076	−	−	−	−	−
	*Pseudomonas aeruginosa* ATCC 27	−	−	−	−	−
	*Klebsiella pneumoniae* NCTC 13438	+	+	+	−	−
Fungi	*Candida albicans* DMSZ 1386	+	+	+	+	−

Clinical case results.

**Table 4:** The antibacterial/antifungal effect of ozonated oils in the literature—(−)—no effect, (+)—low effect, (++)—moderated effect, (+++)—good effect, MDR—Resistent to multiple antibiotics, MRSA—*Staphylococcus aureus* methicillin-resistant, SFO—sunflower oil, OO—Olive oil.

Study	Oil Type	Ozonating Method	PV (mEq O_2_/kg)	Antibacterial/Antifungic Effect	Observations
Puxeddu et al., 2024 [10]	OO/SFO OS Srl (Pesaro, Italy)	-	3110–3520	*C. albicans* (+++/+++) *E. faecalis* (+/++) *E. coli* (−/++) *S. aureus* (++/+) *K. pneumoniae* (−) *P. aeruginosa* (−)	Diffusion method
Moureu et al., 2015 [14]	SFO ± water	50 g oil ±5 g water ≈60 µg/mL, 30 L/h 1–7 h	397 (560–2680)	*S. aureus* (++) *E. coli* (+) *S. uberis* (+)	MIC—Only oil samples ozonated in the presence of water had visible effects
Silva et al., 2020 [11]	OO + SFO (50:50)	100 mL oil 75 µg/mL, 4 L/min 160 min	113.5 ± 3.7	*MRSA* (++)	In vivo: Cutaneous ulceras in mice with diabetes
Song et al., 2018 [15]	Green tea (*Camellia* oil) + ozonated water	-	2000–2200	*S. aureus* (+++) *MRSA* (+++)	In vivo: cutaneous infections in humans
				*S. aureus* (+++) *MRSA* (+++)	In vitro: 400 µL ozonated oil + 50 µL DMSO + 50 µL cult.
				*S. aureus* (17 cm) *MRSA* (13 cm)	Diffusion method (Kirby Bauer)
Grandi et al., 2022 [16]	Liposomal SFO LipozonEye^®^ 10.5%	-	-	*P. aeruginosa MDR* *MRSA* *S. epidermidis* *Streptococcus* spp.	Diffusion method inhibition present at 6–8 h after incubation, but not after 24 h
Montevecchi et al., 2013 [17]	OO Novox^®^	-	-	*S. aureus* (dil. 1:128 = 20.67 ± 0.58 mm) *Porphyromonas gingivalis* (dil. 1:128 = 19 mm)	Diffusion method Oil dilutions 1:2, 1:4, 1:8, 1:16, 1:32, 1:64 si 1:128
Pietrocola et al., 2018 [18]	OO O-zone Gel^®^	-	-	*P. intermedia* (4.5 mm ± 0.38), *A. actinomycetemcomitans* (3.5 mm ± 0.14) *S. mutans* (3.5 mm ± 0.2)	Diffusion method
				*P. intermedia* (10%), *A. actinomycetemcomitans* (10%) *S. mutans* (−)	MIC
Skalska et al., 2009 [7]	SFO	150 mL 20–30 µg/mL 1–50 h	1187	*B. subtillis* (200 mg O_3_/g) *E. coli* (>50 mg O_3_/g) *C. albicans* (>50 mg O_3_/g)	MIC
Zanardi et al., 2013 [19]	Sesame Oil	40 mL oil 45 µg/mL, 1.5 L/min	low: 949 ± 33, medium: 1631 ± 64, high: 3170 ± 101	*S. aureus* *E. faecalis* *P. aeruginosa* *E.coli* *C. albicans*	-
Serio et al., 2017 [20]	SFO	(30% UFS)	335	*E. coli* (22.5 ± 0.07) *P. aeruginosa* (20.75 ± 0.1) *M. luteus* (21 ± 0.28) *S. aureus* (23 ± 0.14)	Diffusion method

## Data Availability

The original contributions presented in this study are included in the article. Further inquiries can be directed to the corresponding author.

## Abbreviations

The following abbreviations are used in this manuscript:

PUFA	Polyunsaturated fatty acids
KI	Potassium iodide
KOH	Potassium hydroxide
NA	Nutrient Agar
SAB	Sabouraud Dextrose medium
MIC	Minimum Inhibitory Concentration
NFkB	Nuclear Factor kappa-light-chain-enhancer of activated B cells
EOO	Extra virgin olive oil
MDR	Resistant to multiple antibiotics
MRSA	Methicillin-resistant *Staphylococcus aureus*
UFS	Sunflower oil
UM	Olive oil
AOAC	Standardized iodometric assay
PV	Peroxide value
MIR	Mid infrared spectroscopy
NIR	Near infrared spectroscopy
HHV	Human herpetic virus
Fe-SOD	Iron superoxide dismutase
Mn-SOD	Manganese superoxide dismutase
Cu-SOD	Copper superoxide dismutase
Zn-SOD	Zinc superoxide dismutase
O_2_	Superoxide radical
DNA	Desoxyribonucleic acid
KatG	Catalase G
KatE	Catalase E
H_2_O_2_	Hydrogen peroxide
ROS	Reactive oxygen species
LPS	Lipopolysaccharides
